# Does Self-Rated Health Predict Both Avoidable and Unavoidable Causes of Mortality?

**DOI:** 10.1093/geroni/igaf122.082

**Published:** 2025-12-31

**Authors:** Ellen Idler, Emily Dore, Courtney Yarbrough

**Affiliations:** Emory University, Atlanta, Georgia, United States; Harvard T.H. Chan School of Public Health, Boston, Massachusetts, United States; Emory University Rollins School of Public Health, Atlanta, Georgia, United States

## Abstract

Self-rated health (SRH) has been known as a predictor of all-cause mortality for decades. The mechanism by which poor SRH is so consistently associated with higher risks of mortality, however, is still unclear. One approach taken to better understand this effect has been to disaggregate mortality by cause of death. This has yielded mixed results, given the sacrifice of sample size for single causes of death, but a benefit has been suggestions that respondents with some knowledge of their risks for, or diagnoses of, such single causes, show enhanced effects of SRH. We introduce the approach of disaggregating all-cause mortality into meaningful groups of causes. Originating in health policy studies, the concepts of preventable and treatable causes of mortality have been used to assess the effectiveness of public health and medical care systems, often by comparing countries. Does SRH have differential effects on causes of mortality that are preventable or treatable, compared with unavoidable causes of death? We analyzed data from the Panel Study of Income Dynamics for 1999-2021, with SRH as a predictor of treatable, preventable (by individual behaviors or by public health measures), preventable-plus-treatable, and unavoidable causes of death (OECD). Results from fully-adjusted competing risks models showed that Poor/Fair/Good SRH compared with Very good/Excellent had significantly increased hazards for treatable (HR 1.99, 95% CI 1.20, 3.32), and for preventable-plus-treatable (HR 1.95, 95% CI 1.57, 2.42) causes of mortality, but not for preventable or unavoidable causes. The findings underscore the mechanism of knowledge of one’s health.

